# Meteorological Variables and Synoptic Patterns Associated with Air Pollutions in Eastern China during 2013–2018

**DOI:** 10.3390/ijerph17072528

**Published:** 2020-04-07

**Authors:** Zhujun Dai, Duanyang Liu, Kun Yu, Lu Cao, Youshan Jiang

**Affiliations:** 1 Key Laboratory of Transportation Meteorology, China Meteorological Administration, Nanjing 210008, China; daizhujun99@163.com; 2 Nanjing Meteorological Bureau of Jiangsu Province, Nanjing 210019, China; mitsuiyk@163.com (K.Y.); jysnjsqxt@163.com (Y.J.); 3 Jiangsu Institute of Meteorological Sciences, Nanjing 210008, China; 4 Nanjing Joint Institute for Atmospheric Sciences, Nanjing 210008, China; 5 Meteorological Observatory of Jiangsu Province, Nanjing 210008, China; caolu36@163.com

**Keywords:** air pollution, synoptic situation pattern, meteorological variables, threshold values

## Abstract

Steady meteorological conditions are important external factors affecting air pollution. In order to analyze how adverse meteorological variables affect air pollution, surface synoptic situation patterns and meteorological conditions during heavy pollution episodes are discussed. The results showed that there were 78 RPHPDs (regional PM_2.5_ pollution days) in Jiangsu, with a decreasing trend year by year. Winter had the most stable meteorological conditions, thus most RPHPDs appeared in winter, followed by autumn and summer, with the least days in spring. RPHPDs were classified into three patterns, respectively, as equalized pressure (EQP), advancing edge of a cold front (ACF) and inverted trough of low pressure (INT) according to the SLP (sea level pressure). RPHPDs under EQP were the most (51%), followed by ACF (37%); INT was the minimum (12%). Using statistical methods and meteorological condition data on RPHPDs from 2013 to 2017 to deduce the thresholds and 2018 as an independent dataset to validate the proposed thresholds, the threshold values of meteorological elements are summarized as follows. The probability of RPHPDs without rain was above 92% with the daily and hourly precipitation of all RPHPDs below 2.1 mm and 0.8 mm. Wind speed, RHs, inversion intensity(ITI), height difference in the temperature inversion(ITK), the lower height of temperature inversion (LHTI) and mixed-layer height (MLH) in terms of 25%–75% high probability range were respectively within 0.5–3.6 m s^−1^, 55%–92%, 0.7–4.0 °C 100 m ^−1^, 42–576 m, 3–570 m, 200–1200 m. Two conditions should be considered: whether the pattern was EQP, ACF or INT and whether the eight meteorological elements are within the thresholds. If both criteria are met, PM_2.5_ particles tend to accumulate and air pollution diffusion conditions are poor. Unfavorable meteorological conditions are the necessary, but not sufficient condition for RPHPDs.

## 1. Introduction

PM_2.5_ is the air particulate matter with an aerodynamically equivalent diameter of less than 2.5 μm [1]. Rising PM_2.5_ levels can lead to worsening air quality, seriously affecting human health. Epidemiological studies have shown associations between ambient air pollution and changes in heart rate variability (HRV) [2,3,4,5]. PM_2.5_ is characterized by the small size and lightweight. It can be transported to some faraway places from its source region, causing a wide range of heavy air pollution [6,7], leading to a wide range of human health hazards [8,9]. As a typical pollutant in heavy pollution weather, PM_2.5_ has become a hot spot of research recently [10,11,12,13]. Beijing–Tianjin–Hebei region [14,15,16,17], the Yangtze River Delta region [18,19,20,21,22] and the Pearl River Delta region [23,24,25] are the three highly air-polluted regions in China. As a result, the incidence of air pollution-related diseases in major cities in these regions has been increasing year by year [8,9,26,27].

Although air pollution is usually linked with human activities, natural processes may also determine noticeable concentrations of hazardous substances in the low atmosphere. Many studies have suggested that continuous air polluting processes are jointly affected by pollutant emissions and the weather conditions that favor the accumulation of pollutants [22,23,28]. Comprehensive analyses of meteorological factors on air pollution were conducted by Zhou [29], who established the statistical model of PM_2.5_ concentrations and meteorological element field. The levels of pollutants may be reduced when emissions can be controlled. However, the impact of meteorological variables on concentrations measured may be marked, and these variables cannot be controlled. If pollutant emission is the internal factor of heavy air pollution, then the meteorological condition should be the external factor [30,31]. Deducing the threshold values of meteorological elements that are conducive to the pollution accumulation is very necessary.

The Yangtze River Delta where Jiangsu Province is located is one of China’s most economically developed regions—and one of the most polluted regions. In this region, many questions have been studied by scientists, such as how meteorological conditions including wind speed affect the concentration of the atmospheric pollutants, how the air pollutants transported from other heavily polluted areas by the synoptic situation patterns and meteorological variables and how changes of atmospheric mixed-layer and temperature inversions affect the air quality. Since most of the past studies are cases studies, they have not provided systematic and comprehensive answers. Moreover, the thresholds of various meteorological elements are not given. Therefore, in order to in-depth knowledge and systematic study the different synoptic situation patterns and meteorological variables on air pollution, heavy air pollution from 2013 to 2018 in Jiangsu Province, China was analyzed in this article. According to the sea level pressure (SLP), the characteristics of the boundary meteorological element distribution are discussed in different seasons, different synoptic patterns and meteorological variables.

The remainder of this paper is organized as follows: Data and methods are described in Section 2. We analyzed synoptic patterns and meteorological conditions associated with heavy PM_2.5_ air pollutions in Section 3. The conclusions are given in Section 4.

## 2. Materials and Methods

### 2.1. Study Area and Data Description

**Figure 1:**
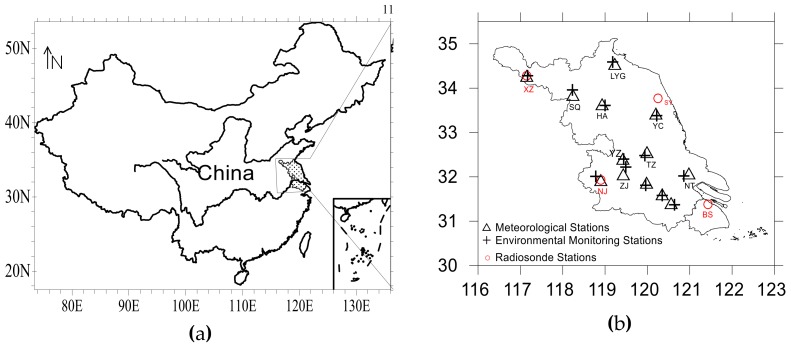
(**a**) The geographical location of Jiangsu province, China (UTC/GMT+08:00); (**b**) The distribution of 13 meteorological, 13 environmental monitoring and 4 radiosonde stations. (Lianyungang: in short LYG; Xuzhou: XZ; Nanjing: NJ; Nantong: NT; Suqian: SQ; Zhenjiang: ZJ; Taizhou: TZ; Huaian: HA; Yancheng: YC; Changzhou: CZ; Suzhou: SZ; Yangzhou: YZ; Baoshan: BS; Sheyang: SY).

The geographical location of Jiangsu Province, China (UTC/GMT+08:00), the distribution of meteorological stations and state-controlled environmental protection stations (SCEPSs) in 13 prefecture-level cities in Jiangsu were shown in Figure 1. In regards to the issue of mismatch between SCEPSs and meteorological stations, the data of SCEPSs nearest to 13 meteorological stations was adopted. According to diurnal variation characteristics and regional differences of heavy PM_2.5_ pollution, 13 cities in Jiangsu Province were divided into four regions: South Jiangsu (Suzhou, Wuxi and Changzhou), Coastal Jiangsu (Lianyungang, Yancheng and Nantong), Southwest Jiangsu (Zhenjiang, Yangzhou, Taizhou and Nanjing) and North Jiangsu (Xuzhou, Huaian and Suqian). Although SCEPSs were distributed in various environments (cities, suburbs, roadsides, parks, etc.), this paper focused on the regional PM_2.5_ heavy pollution, therefore the environmental differences were ignored.

The PM_2.5_ data of Jiangsu Province begins from 2013, therefore the data from 2013–2018 were used in this paper. Meteorological characteristics were discussed by the data from 2013 to 2017 and verified by the data of 2018. The dataset consists of four parts: (1) routine meteorological observation data including the hourly wind speed, wind direction, surface relative humidity (RHs) and precipitation at 13 meteorological stations distributed separately in prefecture-level cities in Jiangsu from 2013 to 2018; (2) the vertical distribution of temperature was obtained from the observational data from four radiosonde stations(Xuzhou, Sheyang, Nanjing and Baoshan) at 8 o’clock. Jiangsu just had three radiosondes, which were respectively Xuzhou, Sheyang, Nanjing, located in the North, Coastal, Southwest Jiangsu. Baoshan, the nearest radiosonde station to South Jiangsu, was located in Shanghai. Hence the data from the four radiosondes may be used to analyze the sounding situation of four regions in Jiangsu. (3) daily reanalysis data with spatial resolution of 2.5° × 2.5° from National Centers for Environmental Prediction (NCEP); (4) hourly PM_2.5_ mass concentration data provided by Jiangsu Environmental Monitoring Center from 2013 to 2018.

Wind speed referred to the horizontal distance of air movement per unit time. Hourly wind speed took 1 second(s) as the time step to calculate the arithmetic average value of the 2minute(min) before the point hour. The sampling frequency of wind speed was 4 times/s.

Wind direction referred to the direction of wind. Hourly wind direction took 1 s as the time step to calculated the vector average value of 2 min before the point hour. The sampling frequency of wind direction was 1 times/s.

Relative humidity referred to the percentage of the actual partial pressure of water vapor in the air and the partial pressure of saturated water vapor under the same conditions. Hourly RHs were calculated as the arithmetic average value at 1 min before the hour. The sampling frequency of RHs was 30 times/min.

Hourly precipitation calculated the cumulative amount of 60 min before the hour. The sampling frequency of precipitation was 1 time/min.

There were four seasons in a year, namely spring (March, April, May), summer (June, July, August), autumn (September, October, November) and winter (January, February, December).

### 2.2. Definition of Heavy Pollution Day

The calculation method for daily average PM_2.5_ concentration was based on “Ambient Air Quality Standards” (GB3095-2012) (Ministry of Environmental Protection of the PRC, 2012b). If the daily average PM_2.5_ concentration above 150 μg m^−3^ was observed in no less than 3 cities of Jiangsu Province in one day, it was defined as a PM_2.5_ HPDs in Jiangsu. If the daily average PM_2.5_ concentration above 150 μg m^−3^ appeared in no less than three cities in a region, it was recorded as a regional PM_2.5_ HPDs (RPHPDs). The pollutant concentrations of the four regions in Jiangsu Province varied greatly, so the definition of RPHPDs was very necessary.

### 2.3. Classification of Synoptic Patterns

**Figure 2:**
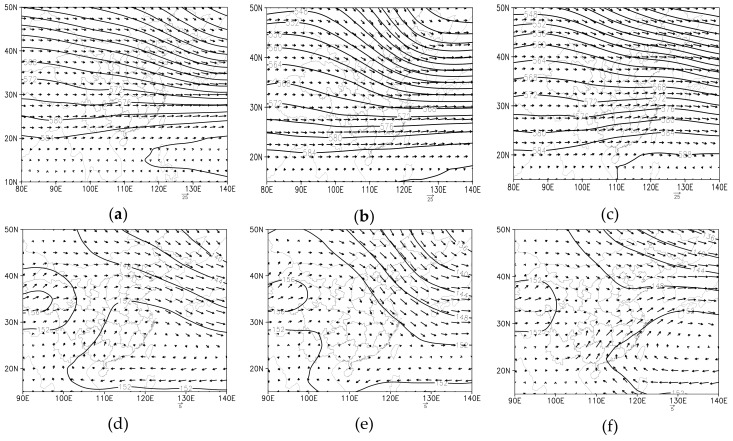

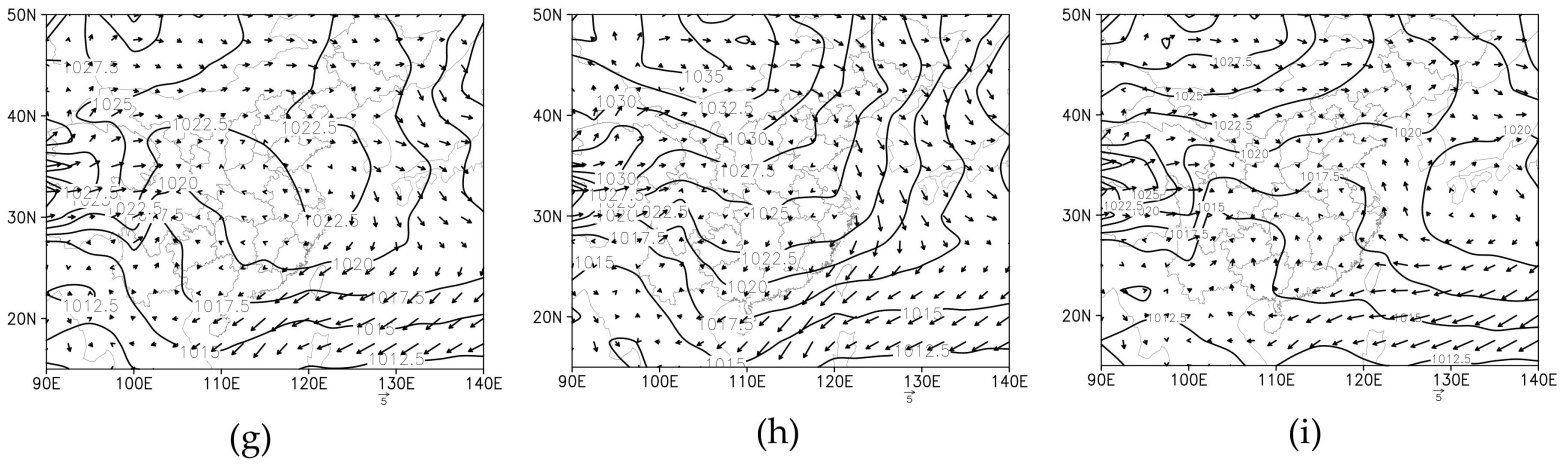
The synoptic situation for each type: equalized pressure (EQP: (**a**,**d**,**g**)), advancing edge of a cold front (ACF: (**b**,**e**,**h**)), inverted trough of low pressure (INT: (**c**,**f**,**i**)); 500 hPa (**a–c**), 850 hPa (**d–f**) and SLP (**g–i**). Among them, contours were pressure field (unit: hPa) and vectors were wind field (unit: m s^−1^).

We analyzed the weather situation, especially SLP, to classified synoptic patterns on RPHPDs (as well as on non-RPHPDs) by the subjective approach, the classification method was the same as Peng, et al. [21] and Huth et al. [32,33,34]. The results showed that, surface conditions on RPHPDs were only dominated by three patterns, respectively were equalized pressure (EQP), advancing edge of a cold front (ACF) and inverted trough of low pressure (INT) (Figure 2).

The three patterns were the necessary external condition for RPHPDs. There were any other synoptic conditions which frequently affect the flow over Jiangsu Province, but RPHPDs only occurred in the context of the three synoptic conditions. Nevertheless, non-RPHPDs were also observed under the three patterns, this was because that the synoptic pattern was the external factors causing RPHPDs [35].

(i) EQP (Figure 2g): when the cold air was blocked in the north, the domain was controlled by equalized pressure;
(ii) ACF (Figure 2h): when the cold air strongly advanced, the domain was controlled by the advancing edge of the cold front;
(iii) INT (Figure 2i): when the domain was controlled by the back of the weak high pressure, the high pressure receded, the inverted trough developed, and the domain was overtaken by the top of the inverted trough.

Upper and lower altitudes under three synoptic flow patterns were described as follows. At 500 hPa, the northwest westerly wind prevailed under EQP; the northwest wind prevailed under ACF; the straight west wind prevailed under INT. That is, in the meridional direction: ACF >EQP>INT (Figure 2a–c). At 850hPa, the west wind prevailed under EQP; the northwest wind prevailed under ACF; the southwest wind prevailed under INT (Figure 2d–f). In the sea-level-pressure field, Jiangsu was on the rear of the high pressure moving towards the sea under EQP; under ACF, Jiangsu was in the wedge at the front of cold high; under INT, Jiangsu was in the uniform pressure field at the front of low pressure. All the wind speeds were very low, and wind directions varied in different regions (Figure 2g–i).

Due to great differences of physical quantities in different seasons, RPHPDs were further classified (Table 1). In terms of four seasons and surface synoptic flow patterns, eight types were obtained: (a) spring INT, (b) summer EQP, (c) autumn EQP, (d) autumn ACF, (e) autumn INT, (f) winter EQP, (g) winter ACF, (h) winter INT.

Considering the contingency of the data when there were less than three RPHPDs, characteristics of meteorological elements were studied only when RPHPDs were above three days in four regions and under different synoptic flow patterns (Table 1). The heavy pollution episodes which met the condition only happen in winter. The requirement was met in all four regions under winter EQP and winter ACF. While for winter INT, it was satisfied in only two regions—Southwest and North Jiangsu.

### 2.4. Method for Calculating MLH

The dry adiabatic curve method was used to calculate the mixing layer height (MLH). The dry adiabatic curve method was proposed by Holzworth [36,37] when studying the average maximum height of the mixing layer in some areas of the United States. As the required data were easy to obtain, the estimation method was widely used in China. The results showed that the dry adiabatic method was more practical than the methods established in the national standard GB/T 3840—91 [38] or Nozaki method [39,40] in calculating the MLH, and it was the most representative method. And its calculated results can denote the actual characteristics of the atmosphere to some extent. The daily maximum height of the mixing layer can be defined as the height below the intersection point of the dry adiabatic line and the temperature profile in the afternoon based on the daily radiosonde data at 0800 LST (Local Standard Time) and the maximum surface temperature data. This method was convenient for areas with radiosonde data and can be used for air pollution potential forecasting. Then, MLHs of Xuzhou, Sheyang, Nanjing and Baoshan radiosonde stations were calculated. Among them, Xuzhou, Sheyang and Nanjing were in North Jiangsu, Coastal Jiangsu and Southwest Jiangsu respectively; Baoshan station was a radiosonde station in Shanghai nearest to South Jiangsu. Thus, these four radiosonde stations were selected to represent the four regions (North Jiangsu, Coastal Jiangsu, Southwest Jiangsu and South Jiangsu) to analyzed the characteristics of MLH in each area.

### 2.5. Method for Calculating Temperature Inversion

Temperature inversion meant that the lower-layer temperature was lower than the higher-layer temperature. The lower height of a temperature inversion (LHTI) was the height (unit: m) nearest the surface when the temperature inversion layer develops. The upper height of a temperature inversion (UHTI) was the height (unit: m) at the top of the temperature inversion layer. The temperature difference in the temperature inversion was the upper temperature of the temperature inversion layer (UTTI) minus the low-level temperature of the temperature inversion layer (LTTI). The height difference in the temperature inversion (ITK) was the height of the LHTI minus the height of the UHTI. The intensity of the temperature inversion (ITI) was the temperature difference divided by the height difference multiplied by 100(unit: °C/100m): ITI = (UTTI − LTTI)/(LHTI − UHTI) * 100.

## 3. Results

### 3.1. Distribution Characteristics of RPHPDs

**Figure 3:**
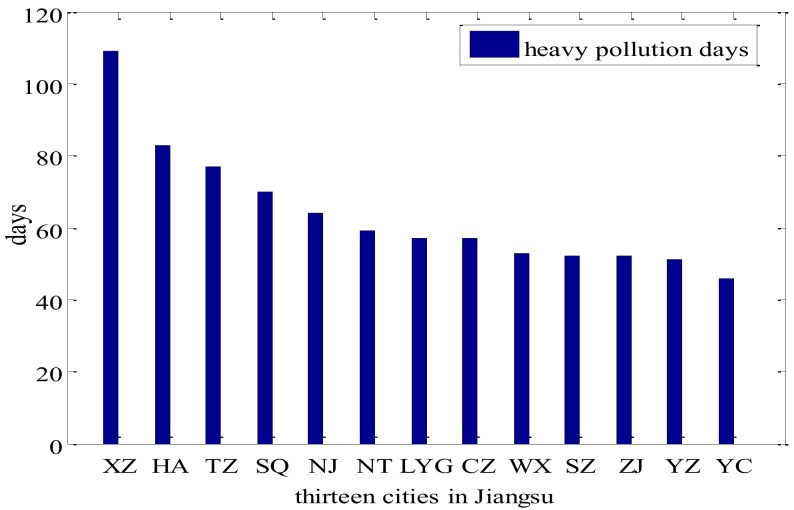
Heavy pollution days of 13 cities in Jiangsu Province, China (unit: days). (Lianyungang: in short LYG; Xuzhou: XZ; Nanjing: NJ; Nantong: NT; Suqian: SQ; Zhenjiang: ZJ; Taizhou: TZ; Huaian: HA; Yancheng: YC; Changzhou: CZ; Suzhou: SZ; Yangzhou: YZ; Baoshan: BS; Sheyang: SY).

Statistical characteristics were obtained by the data from 2013 to 2017 and verified by the data of 2018. PM_2.5_ HPDs in 13 prefecture-level cities of Jiangsu from 2013 to 2017 were shown in Figure 3. The largest number of PM_2.5_ HPDs appeared in Xuzhou (109 days), followed by Huaian (83 days), Taizhou (77 days), Suqian(70 days), Nanjing (64 days), Nantong (59 days), Lianyungang (57 days), Changzhou (57 days), Wuxi (53 days), Suzhou (52 days) and Zhenjiang (51 days); the number in Yancheng was the minimum (46 days).

During 2013–2017, there were a total of 78 RPHPDs in Jiangsu (Table 1), including 33 days in 2013, 17 days in 2014, 17 days in 2015, 7 days in 2016 and 4 days in 2017, that is to say, the number of RPHPDs decreased year by year. Source emissions as a major factor in pollution declined over the years, but were not the focus of this study. This paper analyzed the role of meteorological conditions as objective factors in pollution days, so the threshold value was one of the criteria of pollution intensity. We focused on the local pollution in Jiangsu Province, the transport from other provinces were not taken into account.

According to seasons, the largest number of RPHPDs was observed in winter (65 days), followed by autumn (7 days) and summer (5 days) and the least in spring (1 days).

Considering different synoptic flow patterns, from 2013 to 2017 (Table 1), RPHPDs under EQP was the most (40 days, 51% of the cases), followed by ACF (29 days, 37%); the number under INT was the minimum (9 days, 12%).

### 3.2. Frequencies of RPHPDs under Different Synoptic Flow Patterns

The number of RPHPDs varied in different regions (Table 1). RPHPDs were 37, 26, 43 and 39 in South Jiangsu, Coastal Jiangsu, Southwest Jiangsu and North Jiangsu, respectively. The number of RPHPDs was the largest in Southwest Jiangsu, followed by North Jiangsu; the minimum number was in the Coastal Jiangsu.

The frequency of RPHPDs also varied in different seasons (Table 1). In spring, only one RPHPDs occurred in Southwest Jiangsu in May under INT. In summer, three RPHPDs and two RPHPDs respectively occurred in Southwest Jiangsu and North Jiangsu in June, which may be related to straw burning. In autumn, RPHPDs can occurred in all three patterns, but the probability was low and it only appeared in November. And the occurrence probability under EQP was higher than under ACF and INT. RPHPDs in South Jiangsu, Coastal Jiangsu and Southwest Jiangsu were one day, one day and three days respectively under EQP in autumn, while for ACF and INT in autumn, only one RPHPDs was observed in North Jiangsu and Southwest Jiangsu, respectively. RPHPDs appeared the most frequently in winter, mainly in December and January, followed by February. With regards to the three synoptic flow patterns, RPHPDs occurred more frequently under EQP and ACF and less frequently under INT. Yet no RPHPDs appeared under EQP in February, and no RPHPDs occurred in South Jiangsu under INT in winter.

The frequencies of RPHPDs under the three patterns of EQP, ACF and INT were also different (Table 1). Under EQP, RPHPDs were observed in summer, autumn and winter; under ACF, RPHPDs only occurred in autumn and winter, mainly in winter; under INT, RPHPDs occurred in spring, autumn and winter. The frequency of RPHPDs was high under patterns of g and g2 and low under INT. Moreover, the occurrence of RPHPDs was closely related to the circulation background. Under ACF, the high pressure was in the north and the cold air was active, thus RPHPDs only occurred in autumn and winter. Besides local pollution, large amounts of transportation and rapid accumulation of pollutants were also accompanied under ACF. Therefore, under EQP, the accumulation concentration of local pollutants was relatively high, and the continuous high-pressure control led to lower wind speed and stable stratification, and radiation inversions frequently happened in the night of sunny days. All these conditions finally resulted in the persistent accumulation of pollutants and a high frequency of RPHPDs. As for INT, the uniform pressure field were dominating with low wind speed and stable stratification. Located in the front of low pressure with the prevailing southeast wind, the surface convergence in Jiangsu was strong, which can easily led to the pollutant accumulation, formation of inversion weather, rise of RHs and hygroscopic growth, increasing the occurrence probability of RPHPDs.

Furthermore, frequencies of RPHPDs under eight types varied in four regions (Table 1). For South Jiangsu, the number of RPHPDs for winter ACF were the highest (17 days), followed by winter EQP (15 days), and the minimum number was for autumn EQP (1 day). In Coastal Jiangsu, the number for winter EQP (12 days) were slightly larger than that for winter ACF (11 days), followed by winter INT (2 days) and autumn EQP (1 day). In Southwest Jiangsu, the number for winter ACF (16 days) was larger than that for winter EQP (14 days), followed by winter INT (5 days), summer EQP (3 days) and autumn EQP (3 days), and the minimum number was for spring INT(1 day) and autumn INT (1 day). In North Jiangsu, the number for winter EQP (17 days) was larger than that for winter ACF (14 days), followed by winter INT (5 days) and summer EQP (2 days), and the minimum number was for autumn ACF (1 day). In all four regions, the number for winter EQP or winter ACF was significantly larger than that for other types.

Considering the contingency of the data when there were less than three RPHPDs, winter were not the only season to be considered (Table 1). Winter EQP, winter ACF and winter INT were hereinafter referred to as EQP, ACF and INT for short. Thereby, RPHPDs can be categorized into ten types: EQP in North Jiangsu (EQP_nth), EQP in South Jiangsu (EQP_sth), EQP in Southwest Jiangsu (EQP_sw), EQP in Coastal Jiangsu (EQP_cst), ACF in North Jiangsu (ACF _nth), ACF in South Jiangsu (ACF _sth), ACF in Southwest Jiangsu (ACF _sw), ACF in Coastal Jiangsu (ACF _cst), INT in North Jiangsu (INT_nth) and INT in Southwest Jiangsu (INT_sw).

### 3.3. Variation Characteristics of PM_2.5_ and Meteorological Elements for Ten Types

#### 3.3.1. Concentration of PM_2.5_

**Figure 4:**
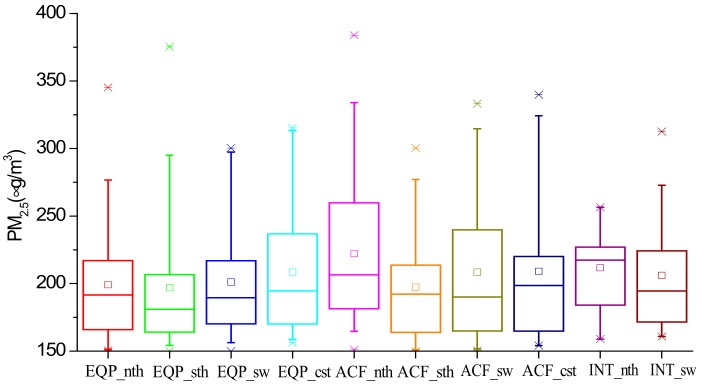
PM_2.5_ concentration distributions of RPHPDs under the ten types. Rectangle: 25%–75% (quartile); rectangle midline: the median; end of the line: 5%–95%; ×: 1% and 99%; □: average. (equalized pressure (EQP), advancing edge of a cold front (ACF) and inverted trough of low pressure (INT).EQP in North Jiangsu (EQP_nth), EQP in South Jiangsu (EQP_sth), EQP in Southwest Jiangsu (EQP_sw), EQP in Coastal Jiangsu (EQP_cst), ACF in North Jiangsu (ACF _nth), ACF in South Jiangsu (ACF _sth), ACF in Southwest Jiangsu (ACF _sw), ACF in Coastal Jiangsu (ACF _cst), INT in North Jiangsu (INT_nth) and INT in Southwest Jiangsu (INT_sw)).

Among the ten types, ACF_nth had the highest values in PM_2.5_ concentration distribution, the high-value range of 25%–75% and the average value (Figure 4). In terms of 25%–75% high-value range and average value, for EQP, the concentration were not the highest in EQP_cst and lowest in EQP_sth; for ACF, the concentration were not the highest in ACF_nth and lowest in ACF_sth. For INT, the concentration in INT_nth was higher than that in INT_sw and the PM_2.5_ concentration in ACF_sth was the lowest among the ten types. In the following section, characteristics of meteorological elements under the ten types were discussed.

#### 3.3.2. Wind Direction and Speed

**Figure 5:**
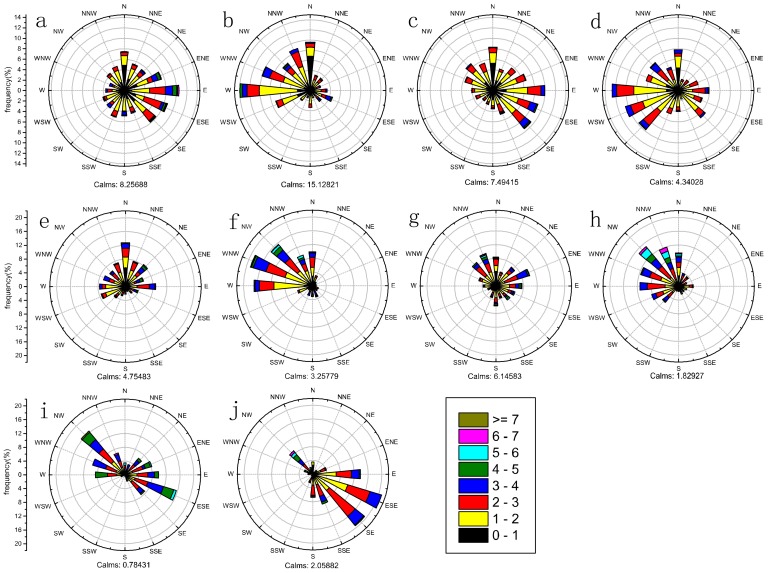
Wind-rose map of flow vector wind in ten types ((**a**) EQP_nth, (**b**) EQP_sth, (**c**) EQP_sw, (**d**) EQP_cst, (**e**) ACF_nth, (**f**) ACF_sth, (**g**) ACF_sw, (**h**) ACF_cst, (**i**) INT_nth, (**j**) INT_sw). The color scale represents the average wind speed during the two minutes before the integral hour point. (equalized pressure (EQP), advancing edge of a cold front (ACF) and inverted trough of low pressure (INT).EQP in North Jiangsu (EQP_nth), EQP in South Jiangsu (EQP_sth), EQP in Southwest Jiangsu (EQP_sw), EQP in Coastal Jiangsu (EQP_cst), ACF in North Jiangsu (ACF _nth), ACF in South Jiangsu (ACF _sth), ACF in Southwest Jiangsu (ACF _sw), ACF in Coastal Jiangsu (ACF _cst), INT in North Jiangsu (INT_nth) and INT in Southwest Jiangsu (INT_sw)).

The prevailing wind direction was different under ten different types (Figure 5). The prevailing wind in EQP_nth was the southeast direction (Figure 5a), the northwest direction in EQP_sth (Figure 5b), the southeast direction in EQP_sw and the southwest direction in EQP_cst, both followed by north direction (Figure 5c,d). In ACF_nth, the most frequent wind direction was north (Figure 5e). For ACF_sth, the direction was northwest (Figure 5f). In ACF_sw, the predominant wind direction was northwest, followed by northeast direction (Figure 5g). In ACF_cst, the wind was northwest (Figure 5h). In INT_nth, the most frequent wind direction was northwest and southeast (Figure 5i). Southeast wind prevails in INT_sw (Figure 5j). Overall, wind directions under EQP in different regions were more dispersed, whereas, under ACF and INT, they were more concentrated. Wind speed under EQP was mostly below 3 m/s. Wind speed under ACF and INT were larger, mainly distributed in 0–5 m/s and 2–5 m/s, respectively.

#### 3.3.3. Diurnal Variation of RHs and Wind Speed

Higher RHs was more likely to cause heavy pollution. It may be caused by the accelerated reaction and hygroscopic growth of particulate matters under high RHs conditions [41], The rise of RH altered the particle size and thus visibility. The wind was an important dynamic factor affecting the diffusion of pollutants in the boundary layer. The wind direction determined the transport direction of pollutants in the atmosphere. Wind speed determined the diffusion and dilution speed of pollutants in the atmosphere, and variations of low-level wind direction and wind speed directly affected the convergence, divergence and concentration distribution of pollutants. Therefore, RHs and wind were important meteorological elements affecting the formation and development of heavy pollution.

**Figure 6:**
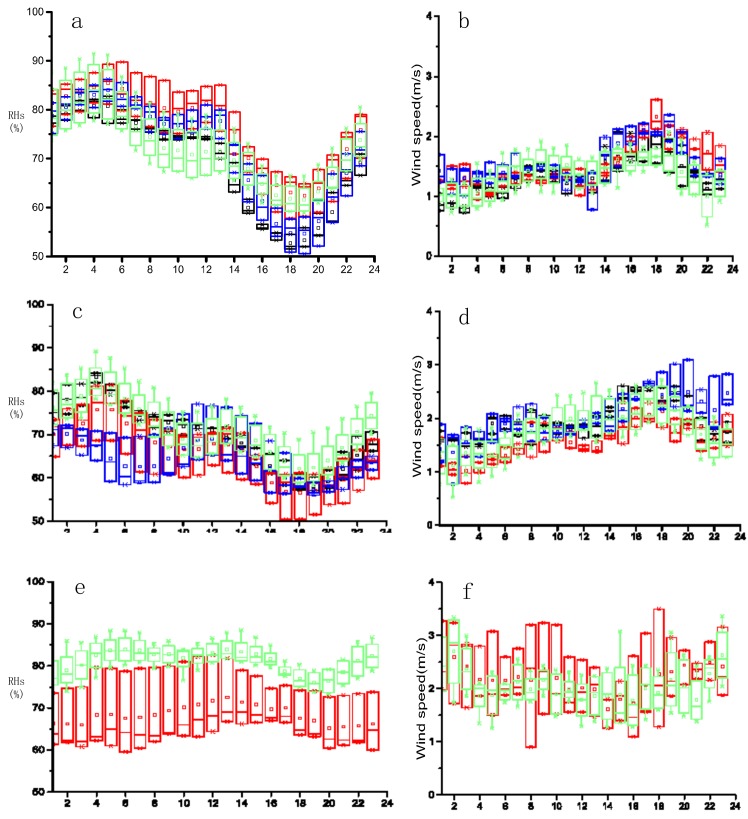
Diurnal variations of wind speed and RHs for EQP(**a**,**b**), ACF(**c**,**d**) and INT(**e**,**f**); red: North Jiangsu, black: South Jiangsu, green: Southwest Jiangsu, blue: Coastal Jiangsu, X-axis was time (unit: h); Rectangle: 25%-75% (quartile); rectangle midline: the median; end of the line: 5%-95%; ×: 1% and 99%; □: average. (equalized pressure (EQP), advancing edge of a cold front (ACF) and inverted trough of low pressure (INT)).

The average diurnal variations of RHs and wind speed were calculated for ten types. Diurnal variations of RPHPDs in different regions were like each other, but there were still some differences (Figure 6). Except for an insignificant negative correlation observed between wind speed and RHs under INT_sw (Figure 6e,f), negative correlation coefficients under the other 9 types were all in the range of 0.77–0.92, passing the 99.9% significance test.

Diurnal variations of RHs and wind speed under EQP and ACF were more significant than INT (Figure 6a–d). The RHs decreased greatly and the wind speed increased notably in the afternoon, reaching a minimum values during 1700–1900 LST. Diurnal variation ranges of RHs and wind speed also varied in different regions. It can be seen that ranges of RHs diurnal variation were larger in Coastal Jiangsu and South Jiangsu than those in North Jiangsu and Southwest Jiangsu. Wind speeds in Coastal Jiangsu and South Jiangsu were slightly larger than that in North Jiangsu for ACF, and the range of RHs diurnal variation in Coastal Jiangsu was the smallest. The wind speed for ACF began to fluctuated and increased at 1600 LST, reaching its peak at 2000 LST and remained at a high level at night.

Compared with EQP and ACF, the diurnal variation of INT was insignificant, and diurnal variation ranges of RHs and wind speed were smaller, but wind speed was larger (Figure 6e,f). RHs in Southwest Jiangsu were significantly larger than North Jiangsu for INT.RHs decreased slightly at 1600 LST and reached the bottom value between 1900 LST and 2000 LST. Wind speed in INT_sw was within 1.8–2.7 m s^−1^ and RHs was within 76%–84% and those for INT_nth were 1.6–2.9 m s^−1^ and 65%–73%, respectively.

### 3.4. Threshold Values of Meteorological Elements Causing RPHPDs

Using statistical methods and meteorological condition data with RPHPDs from 2013 to 2017 to deduce the thresholds, meteorological data of 2018 were used as an independent dataset to validate the proposed thresholds. The threshold values of meteorological elements for RPHPDs were analyzed as follows.

#### 3.4.1. Precipitation

Precipitation rarely occurred in winter on RPHPDs in Jiangsu. There were only three days, two days, one day and three days of precipitation, respectively in South Jiangsu, Coastal Jiangsu, Southwest Jiangsu and North Jiangsu on RPHPDs during 2013–2017. Probabilities of no precipitation on RPHPDs were as high as 92%, 92%, 98% and 92%, respectively. That is to say, the probability of RPHPDs without rain was above 92% with the daily and hourly precipitation below 2.1 mm and 0.8 mm. This was because the wet deposition brought by heavy precipitation can reduce the PM_2.5_ concentration. When the light precipitation occurred on RPHPDs in Jiangsu, RH values increased to 83%–100%, high RH promoted the hygroscopic growth of pollutants [42], which can be explained by the Köhler’s theory [43]. The rise of RH and hygroscopic growth altered the particle size and aggravated the pollutant accumulation [44,45,46].

#### 3.4.2. Wind Speed and RHs

**Figure 7:**
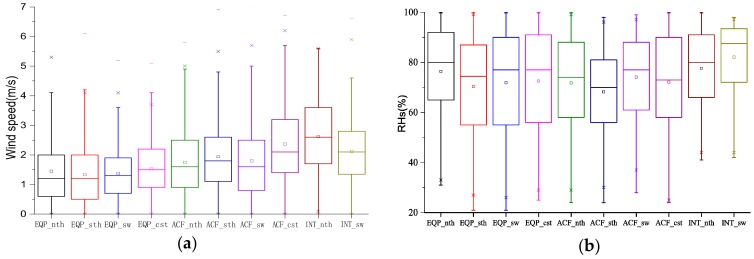
Wind speed (**a**) and RHs (**b**) on RPHPDs (regional PM_2.5_ pollution days) under the ten types. Rectangle: 25%–75% (quartile); rectangle midline: the median; end of the line: 5%–95%; ×: 1% and 99%; □: average.

In this section, the threshold values of wind speed and RHs on RPHPDs were investigated. First, time points of RPHPDs were selected. Then average, maximum and minimum values of wind speed and RHs were calculated. Finally, the threshold values of wind speed and RHs were counted in terms of 25–75% high probability range when RPHPDs occurred. The results were shown in Figure 7. It can be seen from Figure 7a that wind speeds in ten types were very low, with an average of 1.3–2.6 m s^−1^. Wind speeds can reached more than 5 m s^−1^ at some time points in all ten types. In terms of 25%–75% high-value range of RPHPDs, the wind speed varied within 0.5–3.6 m s^−1^. The result was in good agreement with the conclusion of Dai et al. [47]. Specifically, the maximum wind speed was observed in INT_nth, followed by ACF_cst and INT_sw. Wind speeds in INT_nth were mainly within 1.5–3.5 m s^−1^ and the maximum value reaches 7.5 m s^−1^. Average wind speeds in INT_nth, INT_sw and ACF_cst were all above 2 m s^−1^.

The wind speed under EQP was minimum with the average wind speed within 1.3–1.5 m s^−1^ and the wind speed within 0.5–2.3 m s^−1^ in terms of 25%–75% high probability range. Wind speeds in different regions were basically the same. The average wind speed was 1.7–2.4 m s^−1^ under ACF, which slightly higher in South Jiangsu. The wind speed in terms of 25–75% high probability range was within 0.8–2.7 m s^−1^. In a word, the rank of patterns based on the wind speed value on the RPHPDs was INT > ACF > EQP.

The distribution of RHs in RPHPDs was shown in Figure 7b. RHs for ten types varies greatly (21%–100%) with the average RHs within 68%–82%, the maximum was in INT_sw, followed by INT_nth; the minimum was in ACF_sth. Comparing EQP and ACF, INT had the maximum average RHs. It was found that the average RHs under EQP was higher than ACF except in Southwest Jiangsu. RHs on RPHPDs in terms of 25%–75% high probability range was within 55%–92%, with the maximum under INT (65%–92%) and RHs for EQP was basically equivalent to ACF.

To sum up, for INT_nth and INT_sw, the light wind was more likely to cause heavy pollution than the still wind. This was because, under INT, Jiangsu was in the uniform pressure field in the front of low pressure (Figure 2i) and the southeast wind prevails (Figure 5i,j). The light southeast wind was more likely to form a weak wind field convergence near the surface than the still wind. Humidities in INT_nth and INT_sw were also higher than those in other types due to the high RHs of southeast wind. Under EQP, the entire Jiangsu Province was in the center of high pressure (Figure 2g), and wind speeds in four regions were very low. RPHPDs under ACF often occurred before the arrival of cold air moving along the middle or west path (Figure 2h). Dry cold air led to a very low RHs under ACF whose wind speed was slightly higher than that under EQP. Coastal Jiangsu were in the front of the cold front, coupled with the land-sea thermal difference, the wind speed in Coastal Jiangsu was slightly larger than that in other areas.

For the ten patterns, light wind was more likely to cause RPHPDs than still wind. The mechanism was still wind urge pollutants to accumulate in the same place, while light wind prompted pollutants to accumulate in a small area, resulting in weaker vertical shear and vertical mixing of pollutants, which will increased the scope of air pollution. Ultimately, it affected the health of more and more people. The strong wind increased the horizontal vertical shear, and the vertical mixing of pollutants was stronger, which was conducive to the diffusion of pollutants to the upper air. The explanation was consistent with the conclusion of Zhang, et al. [48].

#### 3.4.3. ITI, ITK, LHTI and MLH

Under thermal inversion, especially low-level inversion, the vertical movement and turbulent exchange in the atmosphere were restrained, preventing the vertical transportation and horizontal diffusion of water vapor, smoke and other pollutants in the atmosphere. Thus, it led to persistent heavy pollution. The influence of inversion on the pollution diffusion was related to its ITI, ITK and LHTI [49,50,51,52].

**Figure 8:**
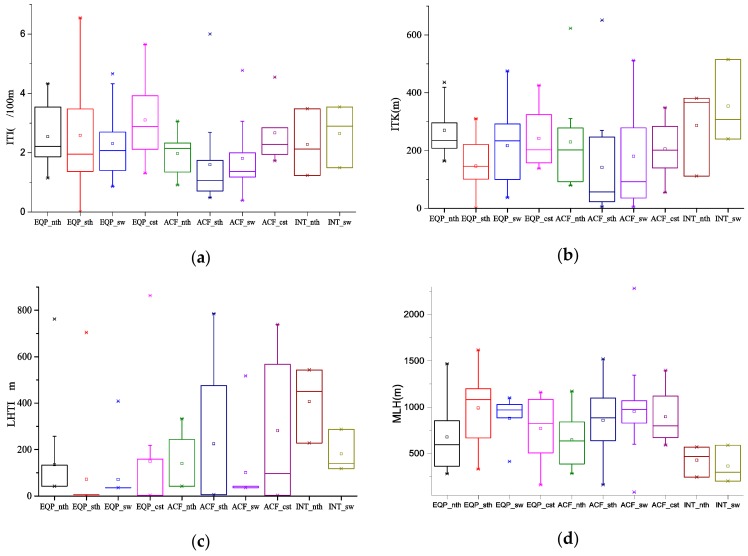
Inversion intensity(ITI) (**a**), height difference in the temperature inversion(ITK) (**b**), the lower height of temperature inversion (LHTI) (**c**) and mixed-layer height (MLH) (**d**) on RPHPDs (regional PM_2.5_ pollution days) under the ten types. Rectangle: 25%–75% (quartile); rectangle midline: the median; end of the line: 5%–95%; ×: 1% and 99%; □: average.

Four radiosonde stations, Xuzhou, Sheyang, Nanjing and Baoshan, were selected as representative stations for North Jiangsu, Coastal Jiangsu, Southwest Jiangsu and South Jiangsu, respectively. The radiosonde data at 0800 LST was used to obtain the characteristics of ITI, ITK and LHTI at the lowest level in ten types of RPHPDs (Figure 8). Statistical results showed that except for type EQP_sth on January 18, 2014, inversion were observed in all RPHPDs. Temperature inversion and heavy pollution were bidirectional feedback mechanisms. A capping inversion was not conducive to the diffusion and convection of pollutants and was easy to form a static and stable weather condition that lead to accumulation of pollution. The occurrence of heavy pollution was often accompanied by temperature inversion. When pollution accumulates to a certain level, aerosol pollution can lead to temperature inversion (Zhong, 2019) [53]. ITI was 0–6.5 °C 100 m ^−1^, with an average of 1.6–3.1 °C 100 m ^−1^. ITK was within 0–651 m, with an average value 142–354 m. LHTI was within 0–1015 m, with an average value 71–407 m. When RPHPDs occurred, ITI, ITK and LHTI were respectively within 0.7–4.0 °C 100 m ^−1^, 42–576 m and 3–570 m in terms of 25%–75% high probability range.

Specifically, the average ITI in EQP_cst was the strongest and that in ACF_sth was the weakest (Figure 8a). Under EQP and ACF, ITI was the strongest in Coastal Jiangsu, and the weakest in Southwest Jiangsu. However, under INT, ITI in Southwest Jiangsu was slightly higher than that in North Jiangsu. For the same region, the average ITI under ACF was lower than that under EQP and INT, and the average ITK under ACF was lower than that under the other two patterns (Figure 8b). Under EQP and ACF, the largest ITK was in North Jiangsu, followed by Coastal Jiangsu, Southwest Jiangsu and South Jiangsu in turn. Under INT, ITK was larger in Southwest Jiangsu than that in North Jiangsu. Only in ACF_sth, ACF_cst and INT_nth, LHTI on a few RPHPDs was relatively higher. LHTI in other types were all below 300 m in terms of 25%–75% high probability range (Figure 8c).

The mixing layer referred to the atmospheric layer with sufficient turbulence below the discontinuous-turbulence interface [54,55,56]. The MLH affected the vertical diffusion of pollutants. Figure 8d showed that the MLH was relatively low (83–2284 m) when RPHPDs occurred. And the average value was less than 1 km (364–993 m). The MLH was within 200–1200 m in terms of 25%–75% high probability range when regional RPHPDs occurred. The MLH was low and the atmospheric dispersion and dilution capability were weak, which was conducive to the accumulation of pollutants. Specifically, the mixing layer was the lowest under INT (INT_nth and INT_sw), and heights were basically the same under EQP and ACF, and MLH were in ranges of 246–740 m, 700–1200 m, 500–1100 m and 202–1100 m in terms of 25%–75% high probability range when RPHPDs occurred in North Jiangsu, South Jiangsu, Coastal Jiangsu and Southwest Jiangsu, respectively.

The ITI, ITK, LHTI and MLH were within 0.7–4.0 °C 100 m^−1^, 42–576 m, 3–570 m, 200–1200 m, respectively. The threshold values of inversion and MLH was coincide with the results of Zhong J.T. [56], Miao Y.C. et al. [57], Huang X. et al. [58] and Ding A. J. et al. [59]. According to Ding A. J. et al. [59], the threshold values ranged extremely, the lower MLH or the greater ITI, the poorer air pollution diffusion condition. Unfavorable meteorological conditions were the necessary but not sufficient condition for RPHPDs.

### 3.5. Reliability Test of Threshold Values

The threshold values were deduced from the winter of 2013–2017, so the heavy pollution data in of 2018 as an independent dataset was used to conduct the reliability tests. In the winter of 2018 consists of January, February and December, with a total of 90 days, there were 29 days which meteorological conditions met both criteria: (1) the pattern was EQP, ACF or INT, (2) the eight meteorological elements were within the thresholds. There were only 11 RPHPDs that occurred in January among the 29 days, accounted for 38%. The proportion was not high, because the meteorological condition was one of factors leading to RPHPDs.

The threshold values of meteorological elements for RPHPDs in Jiangsu in winters of 2013–2017 were tested in Section 3.3. The results (Table 2) show that in the winter of 2018, RPHPDs only occurred in January, and the corresponding synoptic patterns were g and j. The frequency of RPHPDs under EQP was the highest, while only one RPHPDs under INT occurred. The number of RPHPDs in North Jiangsu was the largest, followed by Southwest Jiangsu and South Jiangsu, and no RPHPDs occurred in Coastal Jiangsu. The daily and hourly precipitation of RPHPDs in 2018 was less than 2.1 mm and 0.8 mm. The wind speed, wind direction, RHs, ITI, ITK and LHTI were all distributed within the aforementioned threshold ranges. That is to say, ITI is within 0.7–4.0 °C 100 m ^−1^; ITK was within 42–576 m; LHTI was within 3–570 m; MLHs in North Jiangsu, South Jiangsu and Southwest Jiangsu were also within the range.

After testing, it was found that when the synoptic flow pattern were EQP, ACF or INT and eight meteorological elements were within the threshold ranges, PM_2.5_ particles tend to accumulate, and air pollution diffusion conditions were poor. Unfavorable meteorological conditions were the necessary but not sufficient condition for RPHPDs. For example, in January of 2018, the probabilities of RPHPDs in South Jiangsu, North Jiangsu and Southwest Jiangsu were 2/3, 2/3 and 1/2, respectively, but RPHPDs was not necessarily to occurred as the meteorological condition was only one of factors leading to heavy pollution.

## 4. Conclusions

Continuous air polluting processes were jointly affected by pollutant emissions and the weather conditions that favor the accumulation of pollutants. Steady meteorological conditions were important external factors affecting air pollution. Deducing the threshold values of meteorological elements that were conducive to the pollution accumulation was very necessary to achieve a better control of air pollution. This study provided an in-depth, systematic and comprehensive research about the different synoptic situation patterns and meteorological variables on air pollution, the heavy air pollution from 2013 to 2018 in Jiangsu Province, China.

Surface conditions on RPHPDs were only dominated by three patterns, respectively were equalized pressure (EQP), advancing edge of a cold front (ACF) and inverted trough of low pressure (INT). Most RPHPDs appeared under EQP, followed by ACF, and the last days were under INT. The three patterns were the necessary condition for RPHPDs. There were any other synoptic conditions which frequently affect the flow over Jiangsu Province, but RPHPDs only occurred in the context of the three synoptic conditions. Nevertheless, non-RPHPDs were also observed under the three patterns, since the synoptic pattern was the external factors causing RPHPDs.

The threshold values of meteorological elements when RPHPDs occurred in terms of 25%–75% high probability range were as follows: The daily precipitation was below 2.1 mm; the wind speed was within 0.5–3.6 m s−1; the RHs was within 55%–92%; the thermal ITI was within 0.7–4.0 °C 100 m^−1^; ITK was within 42–576 m; LHTI was within 3–570 m; MLH was within 200–1200 m. The MLH in terms of 25%–75% high probability range when heavy pollution occurred in South Jiangsu, Coastal Jiangsu and Southwest Jiangsu was within ranges of 246–1000 m, 163–1300 m, 162–1200 m and 200–1200 m, respectively. After validation with one-year data, threshold values were proved to be relatively reliable.

We focused on the discussion of meteorological conditions for air pollution diffusion in this paper. the source emissions as a major factor in pollution should be the main solution for air pollution control.

The influence of regional transport was not discussed. In future, if the regional PM_2.5_ concentrations continue to decrease, the threshold values would remain applicable. When the threshold values were met, it represents PM_2.5_ particles tend to accumulate, and air pollution diffusion conditions were poor. Unfavorable meteorological conditions always were the necessary but not sufficient condition for RPHPDs.

We took five-year data to determine threshold values, and only one-year data to finished the reliability test. RPHPDs cases of Jiangsu in 2018 were few and more cases were needed to verify the reliability of threshold values, the combined indicators need to be further constructed. In addition, this threshold needs localization correction when it extended to other regions in China or other regions around the globe. The aim was to provide an easy and quick approach to carry out heavy pollution forecasting and early warning services.

## Figures and Tables

**Table 1:** RPHPDs (regional PM_2.5_ pollution days) during 2013–2017 ^1^. (equalized pressure (EQP), advancing edge of a cold front (ACF) and inverted trough of low pressure (INT)).

Type	South Jiangsu	Coastal Jiangsu	Southwest Jiangsu	North Jiangsu
spring INT	\	\	2014.05.30	\
summer EQP	\	\	2014.06.07, 2014.06.15 2014.06.29	2013.06.14 2013.06.15
autumn EQP	2013.11.07	2013.11.15	2013.11.08, 2013.11.20, 2013.11.21	\
autumn ACF	\	\	\	2016.11.14
autumn INT	\	\	2013.11.09	\
winter EQP	2013.01.12, 2013.01.30, 2013.12.01, 2013.12.02, 2013.12.04, 2013.12.06, 2013.12.24, 2014.01.03, 2014.01.18, 2015.01.08, 2015.01.09, 2015.01.10, 2015.01.11, 2015.12.21, 2015.12.31	2013.12.02, 2013.12.04, 2013.12.24,2014.01.03, 2014.01.18,2014.01.30, 2014.12.29,2014.12.30, 2015.01.04,2015.01.09, 2015.01.10,2015.12.21	2013.01.28, 2013.01.29, 2013.01.30, 2013.12.01, 2013.12.02, 2013.12.06, 2013.12.24, 2014.01.02, 2014.01.03, 2014.01.18, 2014.01.30, 2015.12.31, 2017.01.03, 2017.12.31	2013.01.29, 2013.01.30, 2013.12.04, 2013.12.07, 2013.12.24,2014.01.03, 2014.01.30,2014.12.29, 2015.01.04,2015.01.10, 2015.01.26,2016.01.03, 2016.01.09,2016.12.19, 2016.12.31,2017.01.03, 2017.01.04
winter ACF	2013.01.14, 2013.01.16, 2013.12.03, 2013.12.05, 2013.12.20, 2013.12.25, 2013.12.26, 2014.01.19, 2014.01.20, 2014.02.02, 2015.02.04, 2015.02.12, 2015.02.17, 2015.12.15, 2015.12.23, 2015.12.25, 2016.01.04	2013.12.03, 2013.12.05, 2013.12.25, 2013.12.26, 2014.01.19, 2014.01.20, 2014.02.02, 2014.12.24, 2015.02.04, 2015.12.25, 2016.01.04	2013.01.13, 2013.01.24, 2013.01.26, 2013.02.23, 2013.02.24, 2013.12.03, 2013.12.04, 2013.12.05, 2013.12.15, 2013.12.20, 2013.12.25, 2013.12.26, 2014.01.19, 2015.02.12, 2015.12.15, 2016.01.04	2013.01.08,2013.02.23, 2013.12.03,2013.12.05, 2013.12.15,2013.12.20, 2013.12.25,2014.01.17, 2014.01.19,2014.02.02, 2015.02.12,2015.12.14, 2016.01.04,2016.01.10
winter INT		2013.12.08, 2014.01.31	2013.12.07, 2013.12.08, 2014.01.31, 2015.01.05, 2015.01.24	2013.12.08, 2014.01.31, 2014.02.01, 2015.01.05, 2017.12.23

^1^ The date of RPHPDs,\: No RPHPDs in this type.

**Table 2:** Dates of RPHPDs (regional PM_2.5_ pollution days) and variation range of eight meteorological elements in Jiangsu in the winter of 2018. (equalized pressure (EQP), advancing edge of a cold front (ACF) and inverted trough of low pressure (INT).EQP in North Jiangsu (EQP_nth), EQP in South Jiangsu (EQP_sth), EQP in Southwest Jiangsu (EQP_sw), EQP in Coastal Jiangsu (EQP_cst), ACF in North Jiangsu (ACF _nth), ACF in South Jiangsu (ACF _sth), ACF in Southwest Jiangsu (ACF _sw), ACF in Coastal Jiangsu (ACF _cst), INT in North Jiangsu (INT_nth) and INT in Southwest Jiangsu (INT_sw)).

Weather Types	Date	Daily Precipitation (mm)	Hourly Precipitation (mm)	Wind Speed (m s^−1^)	Humidity (%)	ITI (°C 100 m^−1^)	ITK (m)	LHTI (m)	MLH (m)
EQP_nth	0116, 0117 0118, 0119 0120, 0121 0122, 0129	0.5–2.3	0–0.7	0.1–4.0	60–100	0.6–2.0	5–167	42–686	200–1188
EQP_sth	0101, 0119 0130, 0131	0–0.3	0–0.3	0.1–3.6	50–92	1.5–5.0	10–59	6–676	691–1295
EQP_sw	0119, 0120 0129, 0130	0–0.5	0–0.3	0.1–2.5	50–100	0.5–3.3	22–90	36–63	466–1109
INT_sw	0101	0	0	1.0–4.0	50–100	1.3	308	47	937
